# The Effectiveness of 0.6% Povidone Iodine Eye Drops in Reducing the Conjunctival Bacterial Load and Needle Contamination in Patients Undergoing Anti-VEGF Intravitreal Injection: A Prospective, Randomized Study

**DOI:** 10.3390/jcm8071031

**Published:** 2019-07-13

**Authors:** Michele Reibaldi, Teresio Avitabile, Francesco Bandello, Antonio Longo, Vincenza Bonfiglio, Andrea Russo, Niccolò Castellino, Robert Rejdak, Katarzyna Nowomiejska, Mario Toro, Claudio Furino, Salvatore Cillino, Tito Fiore, Carlo Cagini, Patrizia Grassi, Rosario Musumeci, Clementina Elvezia Cocuzza, Marianna Martinelli, Matteo Fallico

**Affiliations:** 1 Department of Ophthalmology, University of Catania, 95123 Catania, Italy; 2 Department of Ophthalmology, University Vita Salute Hospital San Raffaele, 20132 Milano, Italy; 3 Department of General Ophthalmology, Medical University of Lublin, 20079 Lublin, Poland; 4 Department of Ophthalmology, University of Bari, 70124 Bari, Italy; 5 Department of Experimental Biomedicine and Clinical Neuroscience, Ophthalmology Section, University of Palermo, 90127 Palermo, Italy; 6 Division of Ophthalmology, Department of Surgery and Biomedical Science, University of Perugia, S Maria della Misericordia Hospital, 06129 Perugia, Italy; 7 Laboratory Analysis Unit II, A.O.U. “Policlinico-Vittorio Emanuele”, University of Catania, 95123 Catania, Italy; 8 Laboratory of Clinical Microbiology and Virology, Department of Medicine, University of Milano-Bicocca, 20900 Milano, Italy

**Keywords:** intravitreal injection, conjunctival flora, needle contamination, povidone iodine, endophthalmitis

## Abstract

The study purpose was to assess the efficacy of a preservative-free 0.6% povidone iodine eye drops as perioperative prophylactic treatment for reducing conjunctival bacterial load and the rate of needle contamination in patients undergoing intravitreal anti-vascular endothelial growth factor injection. Enrolled patients were randomized to either the study group (0.6% povidone iodine, three day-prophylactic treatment before the injection) or to the control group (placebo, three day-prophylactic treatment). Conjunctival swabs were obtained before and after the prophylactic treatment in both groups. Intravitreal injections were performed in a sterile fashion. The injection needle and a control needle were collected for microbiological culture. Data from 254 and 253 eyes in the study group and control group, respectively, were analyzed. Bacterial growth from conjunctival swab cultures was significantly lower after 0.6% povidone iodine prophylaxis compared to baseline and to placebo prophylaxis (*p* < 0.001), showing an 82% eradication rate in the study group. No injection needle showed bacterial contamination in the study group, whereas six needles were culture-positive in the control group (*p* = 0.015). No serious ocular and non-ocular adverse events were recorded. The 0.6% povidone iodine solution proved an effective treatment in reducing conjunctival bacterial load and risk of needle contamination.

## 1. Introduction

Endophthalmitis represents the most devastating among ocular complications following intravitreal injection, often leading to blindness.

Antisepsis with topical povidone iodine (PI) has been shown to be the only effective prophylactic method against endophthalmitis [1,2], with the 5% solution preferred over the 10% according to both the European Society of Cataract and Refractive Surgeons (ESCRS) [3] and the American Academy of Ophthalmology (AAO) [4]. Indeed, the 5% solution has been demonstrated to contain more bactericidal free iodine as compared to 10% [3,5].

However, povidone iodine antisepsis does not reduce to nil the risk of endophthalmitis after intravitreal therapy, since its incidence after PI application ranges from to 0.02% to 0.3% [6] and a cumulative rate throughout the treatment series was reported in up to 1% of patients [7].

Studies on conjunctival swab after PI antisepsis showed a significant reduction of bacterial load on the eye surface, but not a complete eradication, with a lowest rate of culture-positive swabs of 3% [8,9,10,11].

Likewise, needles used for intravitreal injections, after povidone iodine antisepsis, have been found to be contaminated by bacteria, with a range varying from 0.4% to 21% [11,12,13,14].

A prophylactic treatment given in the days before the injection, which could allow the bacterial load of the conjunctiva to be reduced, is assumed to strengthen the antiseptic effect of 5% povidone iodine, with a further reduction of the risk of endophthalmitis.

Recently, the ESCRS guidelines speculated on the use of a preservative-free 0.6% povidone iodine eye drops as a prophylactic treatment in the pre-surgery period [3].

According to basic research, <1% povidone iodine solutions have been supposed to present the highest content of bactericidal free iodine [10,15].

The purpose of the present study is to assess the efficacy of a preservative-free 0.6% povidone iodine solution as perioperative treatment for reducing conjunctival bacterial load and the rate of needle contamination in patients undergoing intravitreal injection, as compared to a control group.

## 2. Experimental Section

### 2.1. Study Design

This prospective randomized study was conducted at the Department of Ophthalmology of the University of Catania (Italy), between November 2017 and November 2018, in compliance with the tenets of the Declaration of Helsinki, the Consolidated Standards of Reporting Trials (CONSORT) Statement [16], and the standards of Good Clinical Practice. Institutional ethics committee approval was obtained, and all patients were explained the study objective, methodology, duration, and possible risks before signing their informed consent for participation.

### 2.2. Participants

All consecutive patients scheduled for intravitreal injection treatment were assessed for eligibility. Inclusion criteria were the following: diagnosis of age-related macular degeneration, myopic choroidal neovascularization, diabetic macular edema, or macular edema secondary to retinal vein occlusion; naïve patients scheduled for the first intravitreal injection of anti-vascular endothelial growth factor (VEGF) drugs such as ranibizumab and aflibercept; and age ≥18 years. Exclusion criteria were: previous ocular surgeries except cataract surgery; cataract surgery performed within six months from enrollment; treatment with glaucoma eye drops; administration of any topical antibiotic or steroid within 3 months from enrollment; diagnosis of active eye infection.

After inclusion, enrolled patients were randomly assigned, in a 1:1 ratio, using computer-generated codes, to either the study group or to the control group, in a double-blind manner.

After visit 1 (T0—three days before the injection day), the study group patients instilled in the conjunctival sac of the eye to be operated 1 drop of a topical solution of IODIM^TM^ (ocular drops based on preservative-free 0.6% PI, hyaluronic acid, and medium chain triglycerides, Medivis, Catania, Italy—technical data available as Figure S1, Tables S1 and S2 in Supplementary Material), three times a day for three days, and 1 drop on the injection day (T1), at least 3 h before injection. The control group patients received the same vehicle (ocular drops based on hyaluronic acid and medium chain triglycerides) with the same schedule as the study group.

During the two visits at T0 and T1 a conjunctival swab was obtained for each study eye of the two groups.

All enrolled patients were evaluated at T0 visit (before starting the treatment with IODIM^TM^ or placebo) and at T1 visit, on the day of the injection. Best corrected visual acuity was measured by using Early Treatment Diabetic Retinopathy Study (ETDRS) charts. A complete eye examination, including slit lamp biomicroscopy, Goldmann tonometry, and fundus examination, was performed both at T0 and T1.

### 2.3. Collection of the Conjunctival Sample

From each study eye, two conjunctival swabs were taken at T0 and T1 visit, using the Copan ESwab™ collection device (a tube with 1 mL liquid Amies medium and a FLOQSwab^®^, Copan, Italy). The sterile flocked swab, whose tip is coated with short Nylon^®^ fibers, was applied to bulbar conjunctiva in the infero-temporal quadrant, posteriorly from 3 to 4 mm limbus distance (injection site), was completely rotated over the conjunctiva and, then, collected, paying extremely attention to avoid any contact with lids and lashes and other surrounding structures. If accidental contamination of the conjunctiva occurred, the patient was ruled out. After swabbing procedure, the swab was immediately inoculated onto both a blood agar and a chocolate agar plate [17]. Afterward, the tip of the swab was deposited into Septi-Chek culture broth (Becton Dickinson, Franklin Lakes, NJ, USA) [18].

### 2.4. Injection Procedure and Needle Collection

T2 represented the surgical time-point. All procedures were performed by the same surgeon (Antonio Longo), using facemask and after surgical scrubbing, wearing sterile gown and sterile gloves. Following application of topical 1% tetracaine eye drops, disinfection of periorbital skin, eyebrow, eyelids, and eyelashes was carried out by application of 5% povidone iodine for 5 min. Two eyedrops of 5% povidone iodine were applied to the conjunctival sac from 3 to 5 min before the injection. Sterile draping and sterile lid speculum were placed. Intravitreal injection was performed in the infero-temporal quadrant, 3.5 mm and 4 mm posterior to the limbus in phakic and pseudophakic patients, respectively, by using a 30 gauge needle. Extreme care was taken to avoid any contamination of the needle by contact with eyelashes, surrounding structures or surgeon hands. If this happened, the needle was discarded and not considered for the analysis. After the injection, the needle was removed from the syringe by using a sterile needle holder and placed into Septi-Chek culture broth.

To assess field contamination, for each injection a 30 gauge needle was loaded on a sterile 1 mL syringe and placed uncapped at the surgical field for the same time of the injection needle; afterward, it was collected and cultured. This was denoted the ‘control’ needle in order to differentiate it from the ‘patient’ needle.

### 2.5. Microbiological Determinations

Sheep blood agar plates and chocolate agar plates were cultured, within one hour from collection, at 37 °C for three and seven days, respectively. During incubation, for blood agar plates 5% carbon dioxide atmosphere was used to encourage bacterial growth (aerobic, microaerophilic, and hemolytic bacteria). Anaerobic bags were used to incubate the chocolate agar plates (anaerobic bacteria). Once bacterial growth had been obtained, colony forming units (CFUs) for unique colonies were counted on these culture media.

Each Septi-Chek bottle, containing either a swab or needle sample, was incubated, within one hour from collection, at 37 °C and deemed positive if clouding of the culture media was observed within 5 days. When a Septi-Chek culture sample resulted positive, it was subcultured onto 5% sheep blood agar and chocolate agar to isolate and preliminary identify microorganisms. Antibiotic resistance profile of selected isolated bacterial strains was also investigated by using Kirby-Bauer disc diffusion technique. Microbiologists involved in sample culture and identification of bacteria, were masked with regard to randomization, but were aware of the time-points when samples were obtained.

### 2.6. Adverse Events

Non-ocular and ocular adverse events were recorded. Drop comfort was evaluated in both group by providing each patient with a diary where it was recorded through a 0–10 visual analogue scale (0 = very comfortable; 1–3 = mild discomfort; 4–6 = moderate discomfort; 7–10 = severe discomfort). For each patient the highest value recorded throughout the study period was considered for analysis purpose.

### 2.7. Study Objectives

Mean CFUs value and proportion of broth positive culture from conjunctival swab, and proportion of positive culture from patient injection needle represented the primary outcome measures.

Secondary outcome measures were the rate of adverse events and mean ocular discomfort value.

### 2.8. Statistical Analysis

The sample size (at least 252 eyes for each group) was determined from our preliminary data to detect, with an alpha of 0.05 and a 90% power (two-tailed), a difference of 4% in the rate of positive culture from needles (0.2% and 4.6%, respectively).

Values obtained in two groups were compared using the Student’s *t*-test and the chi-squared test for continuous and categorical variables, where appropriate.

*p*-values lower than 0.05 were considered as statistically significant.

## 3. Results

### 3.1. Patients Disposition and Baseline Characteristics

**Figure 1:**
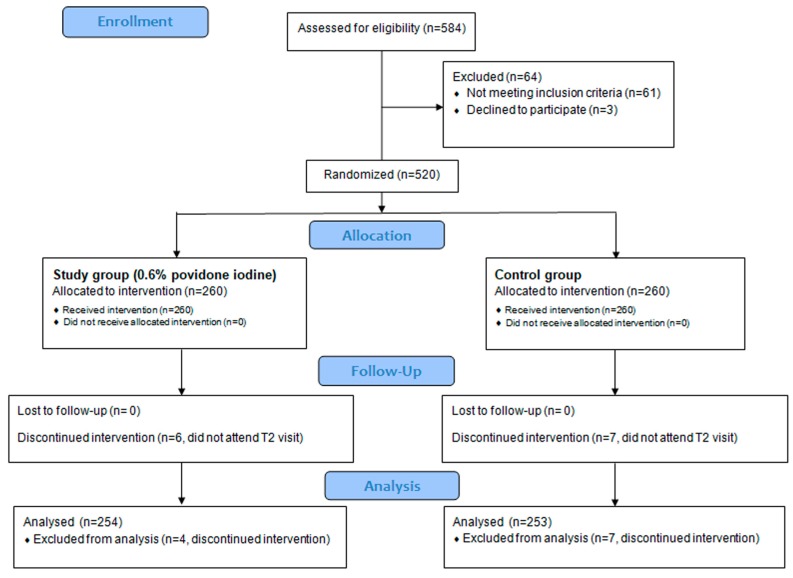
Recruitment flowchart.

Overall, 584 patients were assessed for eligibility, of whom 520 were randomized, of whom 254 and 253 completed the study in the study group and control group, respectively (Figure 1).

Demographic and clinical characteristics of included patients are shown in Table 1. Here, 45% and 48% of patients were male in the study group and control group, respectively (*p* > 0.05). Mean age was 72 ± 8 years in the study group and 74 ± 9 years in the control group (*p* > 0.05). The most common diagnosis was age-related macular degeneration (AMD), appearing in 50% and 53% of cases in the study group and control group, respectively (*p* > 0.05). Diabetic macular edema was present in 39% of patients in the study group and 37% in the control group. Macular edema secondary to retinal vein occlusion (RVO) and myopic choroidal neovascularization (CNV) represented 5% and 4%, and 6% and 6% of cases in the study group and control group, respectively (*p* > 0.05).

### 3.2. Primary Outcome Measures

#### 3.2.1. Microbiological Results from Conjunctival Swab

Blood agar cultures were 254 and 253 at T0 and T1 in the study group and control group, respectively. Likewise, chocolate agar cultures were 254 and 253 at T0 and T1 in the study group and control group, respectively. Table 2 shows CFU results on blood agar and chocolate agar plates at the different time points.

At T0, in the study group and control group respectively, the mean CFU value onto blood agar was 28.3 and 27.8 (*p* = 0.205), and mean CFU value onto chocolate agar was 10.3 and 12.1 (*p* = 0.525).

At T1, in the study group, the mean CFU value on blood agar and chocolate agar significantly decreased to 1.5 and 1.0, respectively. (both *p* < 0.001 vs. T0).

In the control group the mean CFU value on blood agar and chocolate agar was 26.7 and 14.9, respectively, without a significant difference compared to T0 (*p* = 0.806 blood agar T1 vs. T0, *p* = 0.390 chocolate agar T1 vs. T0).

At T1 mean CFU value onto both solid media was also significantly lower in the study group compared to the control group (both *p* < 0.001).

A total of 254 and 253 broth cultures were obtained from conjunctival samples in the study group and control group, respectively. Positive broth cultures were 186 (73%) and 192 (76%) in the study group and control group, respectively, at T0 (*p* = 0.541).

At T1 the number of positive broth cultures was 33 (13%) in the study group (*p* < 0.001 vs. T0) and 187 (74%) in the control group (*p* = 0.682) (between two groups at T1, *p* < 0.001). The eradication rate of bacterial flora in the study group was 82%.

Table 3 displays the results of bacteria growth in positive broth cultures. Overall, the most representative group of bacteria in both groups and throughout the different time-points was the coagulase-negative staphylococci (CoNS), which was isolated in more than 75% of positive cultures followed by *Staphylococcus aureus*. *Streptococci* and other Gram-positive bacteria isolated were also identified at lower rates. With regard to Gram-negative bacteria, their presence was represented only by 3% and 2% of bacterial population between study and control groups and after treatment they were completely eradicated.

**Figure 2:**
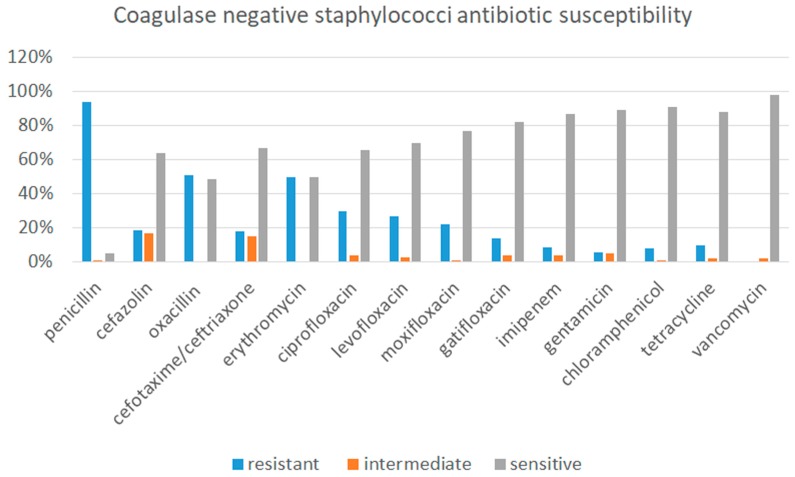
Antibiotic susceptibility of the isolated coagulase negative staphylococci.

Figure 2 shows antibiotic susceptibility of the isolated coagulase negative staphylococci: the highest susceptibility was found for vancomycin, being the largest part sensitive also to gentamicin, chloramphenicol, imipenem and tetracycline. All isolated bacteria other than CoNS resulted susceptible to gentamicin, cefotaxime/ceftriaxone, imipenem, gatifloxacin, and moxifloxacin.

#### 3.2.2. Microbiological Results from Injection Needle

Overall, 254 and 253 patient needles were collected at T2 in the study group and control group, respectively. In turn, 254 and 253 control needles were collected at T2 in the study group and control group, respectively. Of the patient needles, none showed a positive culture in the study group, whereas six needles were culture-positive in the control group (*p* = 0.015). No control needle was found positive for bacterial culture. CoNS strains were isolated in the four out of six positive cultures of the control group. *Staphylococcus aureus* and *Streptococcus viridans* were found in the remaining two positive cultures of the control group.

### 3.3. Adverse Events

No serious non-ocular adverse events were recorded. Overall, 18 and 22 patients experienced headache in the study group and control group, respectively (*p* = 0.515), while 3 and 2 patients were affected by naso-pharyngitis in the study group and control group, respectively (*p* = 1.000). Ocular adverse events are shown in Table 4. No cases of endophthalmitis, retinal detachment, or traumatic cataract were recorded in either of the groups. The largest proportion of patient in both group (95%) did not experience any ocular discomfort, whereas mild discomfort was reported by 12 and 10 patients in the study group and control group, respectively (*p* = 0.828), and severe discomfort in 1 and 2 patients in the study group and control group, respectively (*p* = 1.000) (Table 5).

## 4. Discussion

The present randomized clinical trial proved the efficacy of pre-injection treatment with 0.6% povidone iodine eye drops in reducing the proportion of specimens positive for bacterial growth both from conjunctival swabs and post-injection needles, compared to a control group.

Povidone iodine is the most widely used antiseptic and disinfectant agent for pre-operative preparation in eye surgery, being the 5% concentration recommended by the guidelines of the ESCRS and AAO [3,4].

Nonetheless, endophthalmitis after intravitreal procedure does occur despite povidone iodine disinfection, with an incidence of up to 0.3% of cases [6]. This figure increases to 1% when it comes to the cumulative rate over the treatment period with multiple injections [7].

Considering the ever-increasing number of intravitreal injections performed routinely, with over 20 million intravitreal injections estimated in 2016 worldwide [19,20], and the severely impaired visual outcome following endophthalmitis [6], the introduction of a prophylactic treatment in the days before the injection which could reduce the risk of EO would be of great clinical relevance.

It has been largely demonstrated that prophylaxis with topical antibiotics does not reduce the risk of endophthalmitis after intravitreal injection, being instead associated with a higher incidence of such event [21] and greater antibiotic resistance [22].

Several in vitro studies proved the antimicrobial efficacy of ophthalmic solution containing 0.6% povidone iodine, which showed a faster bactericidal activity compared to traditional 5% PI solution [23,24]. The greater bactericidal efficacy of 0.6% PI compared to more concentrated solution has been related to the increased availability of free iodine, which is the active antimicrobial component, in lower concentration solution [15]. The highest value of the concentration of free iodine is 0.1% [25]. According to the basic research, <1% povidone iodine solutions have been supposed to present the highest content of bactericidal free-iodine [10,15]. On the other hand, Trost et al.’s study bets on a safe, bactericidally effective concentration of 0.05–0.5% for ocular tissues [26]. From these studies, we selected 0.6% povidone iodine in this study. It was estimated that with 0.6% povidone iodine, it would be diluted at the eye surface and approach 0.1%.

Additionally, more diluted povidone iodine solutions have been supposed to present a better tolerability compared to 5% PI solution since sever corneal epithelial damage demonstrated after 5% povidone iodine application is lower for 2.5% PI solution and no longer present at 1% and 0.5% concentration [27].

The antimicrobial efficacy of more diluted PI solution has been demonstrated by numerous clinical studies as well [28,29,30,31].

Peden et al. reported their data on more than 35,000 intravitreal injections performed after disinfection with different concentration of povidone iodine, showing that disinfection with dilute PI solution represents a valid alternative to standard 5% solution in betadine-sensitive patients. Moreover, no cases of endophthalmitis were found in the group receiving 0.6% PI disinfection [32].

Pepose et al. conduced a randomized clinical trial on the efficacy and safety of a suspension containing 0.6% PI and 0.1% dexamethasone in adenoviral conjunctivitis. This suspension resulted safe, well tolerated, and clinically effective [33].

Therefore, we decided to assess the effectiveness of a treatment with preservative-free 0.6% povidone iodine eye drops, given three times a day for three days before the intravitreal injection, to enhance the routine 5% PI disinfection.

Since more than 90% of bacteria inoculations occur at the moment of the intravitreal injection because of needle contamination with conjunctival surface, reducing the conjunctival bacterial load allows us to reduce the risk of infection [34].

In our trial, only patients undergoing intravitreal injection of anti-VEGF agents were included, whereas intravitreal dexamethasone implant was not considered due to different caliper of needle applicator and differences in drug characteristics [35,36].

Our findings showed that 3-day pre-injection treatment with 0.6% PI reduced by 82% the number of bacterial growth-positive swab cultures, decreasing from 74% at the baseline to 14% after prophylaxis. Furthermore, CFUs over the culture plates were significantly reduced after the 0.6% PI treatment.

The results from post-injection needle cultures corroborated the conjunctival swab culture findings, showing no cases of bacterial growth in the 0.6% PI group and 2% of needles positive for bacterial growth in the control group. No control needles were found positive for bacterial growth, showing a flawless methodology.

In the present study, the proportion of post-injection needles with positive bacterial growth in the control group, after routine 5% povidone iodine disinfection, was 2%. Likewise, De Caro et al. [11] reported in their studies a rate of post-injection needle contamination of 2% after 5% povidone iodine application. Nentwich et al. [13] found positive bacterial growth in 0.4% of post-injection needles, after application in the conjunctival sac of 1% povidone iodine. The findings of Stewart et al. and Tufan et al. are different compared to the prior results, since 18% and 21% of post-injection needles showed positive bacterial growth, respectively [12,14]. A possible reason for this difference could be related to difference in methodologies.

According to our results, the most common microorganism group isolated from both conjunctival swab and post-injection needle culture was the coagulase negative staphylococci (CoNS) including the most representative *Staphylococcus epidermidis*, which is consistent with literature reports.

Furthermore, the preservative-free 0.6% povidone iodine solution proved to be well tolerated with no safety concerns: there were no serious systemic adverse events in either groups and no difference in ocular adverse events were found between the study group and control group.

No cases of endophthalmitis were found in either of the groups. Considering that the population of each group was slightly more than 250 eyes, this finding is in accordance with the incidence reported in literature, ranging between 0.02% and 0.3% [6]. Additionally, the present trial was not powered enough to show a difference in endophthalmitis rates between the two groups. Our purpose was to assess whether the treatment with 0.6% PI eye drops was effective in reducing the conjunctival bacterial load before the intravitreal injection and the post-injection needle contamination. This is relevant also from a clinical point of view, since it has been clearly demonstrated that the pathogenic mechanism of post-injection endophthalmitis is mostly based on direct inoculation of bacteria from conjunctival surface into the vitreous chamber at the very moment of the intravitreal injection [34].

The following limitations characterized the present study. First, the efficacy of the treatment with 0.6% PI eye drops was evaluated by analyzing difference in bacterial growth from conjunctival swabs and post-injection needles. The best method to assess it would have been to compare the incidence of endophthalmitis between the study group and a control group. However, a randomized clinical trial aimed to assess the efficacy of such a prophylactic treatment in reducing the incidence of endophthalmis following intravitreal treatment is unlikely due to the rarity of the event and the subsequent excessively large power of the study. Second, there is the possibility that the enrolled patients had not been compliant with the treatment schedule provided. However, each patient was given a diary to record administration time, eye discomfort, and complications. According to the data, all patients proved compliant with the assigned treatment schedule.

In conclusion, the present randomized clinical trial provided evidence on the effectiveness of the treatment with 0.6% povidone iodine eye drops, administered 3 days before the intravitreal injection, also showing a good safety profile. This prophylactic treatment allows us to reduce the bacterial load on the conjunctiva, enhancing the disinfectant activity of 5% povidone iodine. As a consequence, the risk of endophthalmitis is supposed to be further reduced.

## Figures and Tables

**Table 1:** Characteristics of study population.

	Study Group (*n* = 254)	Control Group (*n* = 253)
Male *n*, %	114, 45%	121, 48%
Age (mean ± SD)	72 ± 8	74 ± 9
**Diagnosis**		
AMD	126 (50%)	135 (53%)
DME	100 (39%)	94 (37%)
RVO	12 (5%)	10 (4%)
Myopic CNV	16 (6%)	14 (6%)
**Agent**		
Ranibizumab	121 (48%)	119 (47%)
Aflibercept	133 (52%)	134 (53%)

Footnote: SD: standard deviation; AMD: age-related macular degeneration; DME: diabetic macular edema; RVO: retinal vein occlusion; CNV: choroidal neovascularization.

**Table 2:** Colony forming units on blood agar and chocolate agar plates.

	Blood Agar				Chocolate Agar			
	T0		T1		T0		T1	
*N* Units	Study Group	Control Group	Study Group	Control Group	Study Group	Control Group	Study Group	Control Group
0 to 10	172	181	240	168	209	203	246	197
11 to 100	74	61	14	76	41	48	8	51
101 to 1000	8	11	0	9	4	2	0	5
Mean	28.3	27.8	1.5	26.7	10.3	12.1	1	14.9
SD	45.4	51.6	6.4	48.9	30.6	32.9	5.8	39.8
Tot	254	253	254	253	254	253	254	253

Footnote. SD: standard deviation.

**Table 3:** Isolated bacteria from the conjunctival swab.

	T0		T1	
	Study Group	Control Group	Study Group	Control Group
Positive broth culture	186 (73%)	192 (76%)	33 (13%)	187 (74%)
Coagulase-negative *Staphylococcus*	143 (77%)	157 (82%)	27 (82%)	146 (78%)
*Staphylococcus aureus*	13 (7%)	10 (5%)	2 (6%)	15 (8%)
α-Hemolytic *Streptococcus*	5 (3%)	3 (2%)	1 (3%)	5 (3%)
Β-Hemolytic *Streptococcus*	2 (1%)	4 (2%)	1 (3%)	3 (2%)
*Streptococcus* group D	6 (3%)	3 (2%)	0	4 (2%)
*Corynebacterium* species	6 (3%)	6 (3%)	0	3 (2%)
*Propionibacterium* acnes	2 (1%)	4 (2%)	1 (3%)	4 (2%)
*Micrococcus* species	2 (1%)	3 (2%)	0	4 (2%)
Other gram-negative rods	5 (3%)	4 (2%)	0	3 (2%)
*Bacillus* species	4 (2%)	2 (1%)	1 (3%)	3 (2%)

**Table 4:** Rate of ocular adverse events.

	Study Group (*n* = 254)	Control Group (*n* = 253)	Kruskall-Wallis
Conjunctival hyperemia *n*, (%)	2 (0.8%)	2 (0.8%)	*p* > 0.05
Conjunctival discharge *n*, (%)	2 (0.8%)	3 (1.2%)	*p* > 0.05
Conjunctival follicles/papillae *n*, (%)	1 (0.4%)	1 (0.4%)	*p* > 0.05
Eye pain *n*, (%)	0	0	*p* > 0.05
Corneal epithelial erosion *n*, (%)	3 (1.2%)	1 (0.4)	*p* > 0.05
Keratitis *n*, (%)	0	0	*p* > 0.05
Eyelid edema *n*, (%)	0	0	*p* > 0.05

**Table 5:** Eye discomfort assessment in the two group throughout the study period.

	Study Group (*n* = 254)	Control Group (*n* = 253)	Kruskall-Wallis
No discomfort *n*, (%)	241 (95%)	241 (95%)	*p* > 0.05
Mild discomfort *n*, (%)	12 (5%)	10 (4%)	*p* > 0.05
Moderate discomfort *n*, (%)	1 (0.4%)	2 (0.8%)	*p* > 0.05
Severe discomfort *n*, (%)	0	0	*p* > 0.05

## Supplementary Materials

The following are available online at https://www.mdpi.com/2077-0383/8/7/1031/s1.
