# Supplementary material for: The Effectiveness of 0.6% Povidone Iodine Eye Drops in Reducing the Conjunctival Bacterial Load and Needle Contamination in Patients Undergoing Anti-VEGF Intravitreal Injection: A Prospective, Randomized Study
Source: J Clin Med. 2019 Jul 13;8(7):1031. doi: 10.3390/jcm8071031 (PMC6678890; doi:10.3390/jcm8071031)
Supplement: Supplementary file 1 [file jcm-08-01031-s001.pdf]

GB Sterile eye drops
10 ml amber glass bottle with dropper and screw cap

INSTRUCTIONS FOR USE

COMPOSITION

Medium-chain triglycerides (MCT), Sodium hyaluronate, Glycerol, Vitamin E TPGS, Potassium citrate, Sodium chloride, Citric acid monohydrate, Povidone-iodine 0,6%, Purified water.

PACKAGE

IODIM® 10 ml amber glass bottle with dropper and screw cap, containing sterile eye drops, supplied in a cardboard box.

CHARACTERISTICS AND HOW IT WORKS

IODIM® ophthalmic solution is supplied as a sterile isotonic solution, lipid based due to the presence of medium-chain Triglycerides (MCT). It also contains Sodium Hyaluronate (hydrophylic polymer), Glycerol (moistening agent), Vitamin E TPGS, Mineral salts and Povidone-iodine (preservative).

IODIM® contains MCTs, which interact with the outer lipid layer of the tear film and form a protective film that impedes the hyper-evaporation of the watery component of the tears; it contains sodium hyaluronate, which interacts mechanically with the mucinic layer of the tear film, retains the water and helps keep the surface of the eye stable for a long period of time. In fact, 2 instillations a day are sufficient to produce a feeling of comfort and to improve the condition of the eye surface. **IODIM®** also contains glycerol, which has a moistening effect, mineral salts (sodium, potassium, chloride and citrate), which have a nutritional function of the surface of the eye, besides regulating the pH and tear osmolarity, Vitamin E TPGS, which favours the wettability of the eye surface and Povidone-iodine, which ensures the sterility of the solution before and after opening the bottle.

INDICATIONS

IODIM® stabilises the tear film and defends the surface of the eye in all cases of tear film disorders, present also in patients affected by recurrent eye infections.

DOSAGE AND HOW TO USE

Remove the plastic safety seal on the neck of the bottle (Figure 1) and unscrew the cap (Figure 2).

- Tilt your head slightly back and pull down the lower lid of your eye with your index finger to create a small pocket. Hold the dropper vertically with the tip pointing down above the eye (Figure 3).
- Look upward and squeeze lightly to instill 1 or 2 drops into the eye (Figure 4), 1 or 2 times a day, according to the doctor's instructions, then close your lids delicately for a few moments to ensure that the solution is evenly distributed over the surface of the eye.

STORAGE AND EXPIRATION

- Do not use the product after the expiration date marked on the box. Keep in the refrigerator at a temperature of between 2°C and 8°C.
- Keep away from light inside the cardboard box.
- Use within 28 days of opening, after which any remaining product must be discarded.

PRECAUTIONS/WARNINGS

- Wash your hands before using
- The product is for ophthalmic use only
- The product must be used by one patient only
- Do not use the product if, when opening for the first time, the packaging is open or appears to have been tampered with
- Keep out of reach of children
- Do not use if specifically hypersensitive to any of the components
- Do not injest the solution
- To avoid any contamination, do not touch any surfaces with the dropper and avoid any direct contact with the eyes
- If any undesired effects occur suspend the treatment and consult a doctor
- Do not instill simultaneously with other ophthalmic products because this might alter its effects; wait for at least 15 minutes in between instillations
- Do not use the product after the expiration date on the packaging
- Do not use with your contact lenses on
- Close the bottle well after use
- The expiration date is valid if the product is whole and properly stored
- Use in adults
- After instillation of the **IODIM®** product may occur a transient burning sensation or pricking

Figure S1: IODIM technical data.

IODIM
Long term stability (2-8°C)
Batch n. SS049
Manufacturing: 08/02/2016
Start of stability: 23/02/2016

Table S1: IODIM long-term stability characteristics.

**TEST**	**SPECIFICATIONS**	**Time (months)**							
		**0**	**1**	**3**	**6**	**9**	**12**	**18**	**24**
**Appearance**	Clear solution of brown color	Conform	Conform	Conform	Conform	Conform	Conform	Conform	Conform
pH	5.5-5.8	5.7	5.7	5.7	5.6	5.7	5.6	5.6	5.7
Osmolality	(250-320) mosmol/kg	287	286	287	287	282	285	287	290
Content of PVP iodine	0.66-0.60 (%)	0.64	0.64	0.63	0.63	0.63	0.64	0.61	0.60
Sterility	Conform to EP	Conform	N/A	N/A	Conform	N/A	Conform	N/A	Conform

In-use stability (2-8 °C)
Batch n. SS049
Manufacturing: 08/02/2016

Table S2: IODIM 0-8 weeks stability characteristics.

Tests/Analysis time (weeks)	Specifications	0 (R)	14 days	4 weeks (A)	4 weeks (R)	6 weeks	8 weeks (A)	8 weeks (R)
Appearance	Clear solution, brown color	Conform	Conform	Conform	Conform	Conform	Conform	Conform
pH	5.5-5.8	5.6	5.7	5.7	5.7	5.7	5.7	5.7
Osmolality	250-320 mosmol/kg	287 mosmol/kg	288 mosmol/kg	284 mosmol/kg	284 mosmol/kg	290 mosmol/kg	286 mosmol/kg	285 mosmol/kg
Content of PVP iodine	0.66-0.60 (%)	0.63 mg/ml	0.62 mg/ml	0.62 mg/ml	0.62 mg/ml	0.63 mg/ml	0.63 mg/ml	0.63 mg/ml
Sterility	Conform to EP	Complies	N/A	N/A	N/A	N/A	Complies	Complies

Start of stability: 17/02/2016
R: Reference bottles; A: Actuated bottle
